# Changes in heart morphometric parameters over the course of a monocrotaline-induced pulmonary arterial hypertension rat model

**DOI:** 10.1186/s12967-020-02440-7

**Published:** 2020-06-30

**Authors:** Mateusz K. Hołda, Elżbieta Szczepanek, Joanna Bielawska, Natalia Palka, Dorota Wojtysiak, Paulina Frączek, Michał Nowakowski, Natalia Sowińska, Zbigniew Arent, Piotr Podolec, Grzegorz Kopeć

**Affiliations:** 1 HEART-Heart Embryology and Anatomy Research Team, Department of Anatomy, Jagiellonian University Medical College, Kopernika 12, 31-034 Kraków, Poland; 2 Department of Cardiac and Vascular Diseases, Jagiellonian University Medical College, Kraków, Poland; 3 Division of Cardiovascular Sciences, The University of Manchester, Manchester, UK; 4 Manor Veterinary Clinic, Folkestone, UK; 5 Department of Animal Genetics, Breeding and Ethology, University of Agriculture in Cracow, Kraków, Poland; 6 Department of Clinical Oncology, University Hospital, Kraków, Poland; 7 Center of Experimental and Innovative Medicine, University Center of Veterinary Medicine JU-AU, University of Agriculture in Cracow, Kraków, Poland

**Keywords:** Pulmonary hypertension, Monocrotaline-induced PAH, Cardiac remodelling, Left ventricle mass loss, Right ventricular failure

## Abstract

**Background:**

Aim of this study was to assess changes in cardiac morphometric parameters at different stages of pulmonary arterial hypertension (PAH) using a monocrotaline-induced rat model.

**Methods:**

Four groups were distinguished: I–control, non-PAH (n = 18); II–early PAH (n = 12); III–end-stage PAH (n = 23); and IV–end-stage PAH with myocarditis (n = 7).

**Results:**

Performed over the course of PAH in vivo echocardiography showed significant thickening of the right ventricle free wall (end-diastolic dimension), tricuspid annular plane systolic excursion reduction and decrease in pulmonary artery acceleration time normalized to cycle length. No differences in end-diastolic left ventricle free wall thickness measured in echocardiography was observed between groups. Significant increase of right ventricle and decrease of left ventricle systolic pressure was observed over the development of PAH. Thickening and weight increase (241.2% increase) of the right ventricle free wall and significant dilatation of the right ventricle was observed over the course of PAH (p < 0.001). Reduction in the left ventricle free wall thickness was also observed in end-stage PAH (p < 0.001). Significant trend in the left ventricle free wall weight decrease was observed over the course of PAH (p < 0.001, 24.3% reduction). Calculated right/left ventricle free wall weight ratio gradually increased over PAH stages (p < 0.001). The reduction of left ventricle diameter was observed in rats with end-stage PAH both with and without myocarditis (p < 0.001).

**Conclusions:**

PAH leads to multidimensional changes in morphometric cardiac parameters. Right ventricle morphological and functional failure develop gradually from early stage of PAH, while left ventricle changes develop at the end stages of PAH.

## Background

Pulmonary arterial hypertension (PAH) is a rare disease that is characterized by elevated blood pressure in the pulmonary arteries and is caused mainly by progressive remodeling of the distal pulmonary arterioles, which increases vascular resistance [1]. Pulmonary hypertension gradually leads to increases in right ventricular afterload, thus contributing to the development of pressure-overload-induced right ventricle dysfunction and further premature death due to severe heart failure [2]. Development of right ventricular heart insufficiency, secondary to PAH, is a negative predictive factor [3].

Recent years have seen advances in the understanding of the pathobiology of PAH and its natural history, prognostic indicators, and therapeutic options; however, many important questions remain unanswered [4]. Clinical and experimental studies show that over the course of PAH, opposing changes occur in both heart ventricles. The volume of the right ventricle increases, whereas the left ventricle’s volume decreases, and there is also a reduction of stroke volume, and ejection fraction of both ventricles [5]. Individual pieces of data from clinical studies and those conducted on animal models also indicate decreases in left ventricle mass [6]. Although mechanisms underlying right ventricle hypertrophy and right ventricular failure are fairly well known, remodeling of the left ventricle and its mechanisms are poorly understood [7].

PAH is mostly diagnosed in its advance stage because of the nonspecific nature of early symptoms and signs, which may not be noticeable for months or even years [8]. A thorough evaluation of a patient is critical to correctly characterize the stage of PAH and to prevent its rapid progression as well as to minimize complications. Cardiac studies, including echocardiography and right heart catheterization, are key elements in this assessment [9].

Finding specific changes in dimensions of the heart over the course of PAH may improve the diagnosis and treatment process and contribute to preventing or delaying PAH complications [10]. Moreover, this knowledge may also help to better understand the pathophysiology of PAH and heart failure. For example, left ventricular hypotrophy in patients with PAH is considered as a potential mechanism of pulmonary edema in the course of restoration of the pulmonary flow after lung transplantation.

Nevertheless, currently, little is known about the evolution of heart-related dimensions over the natural course of PAH. Therefore, we aimed to investigate changes of cardiac morphometric parameters at different stages of PAH using a monocrotaline rat model.

## Methods

### Animal model

This study was approved by the 2nd Local Ethical Committee in Cracow, Poland (No 60/2016), and was performed in accordance with European Union directives on the care and use of experimental animals. This study was performed using Wistar rats (Experimental Medicine Center of the Medical University of Bialystok, Poland). The animals were kept in standard, controlled conditions with a temperature of 22 ± 2 °C, a 12:12 h light-darkness cycle, and with free access to water and food. A total of 66 Wistar male rats (8 weeks old) were randomly assigned to two groups after being quarantined for 2 weeks, and the date of assignment was designated as day 0. In the study group (monocrotaline-induced PAH model, n = 48), animals were injected intraperitoneally with a single dose of 60 mg/kg of monocrotaline (Sigma Aldrich, Germany) with Dulbecco’s Phosphate Buffered Saline (3 mL/kg) medium (Sigma Aldrich, Germany) to induce PAH [11]. In the control group (non-PAH, n = 18), rats were injected intraperitoneally with Dulbecco’s Phosphate Buffered Saline (3 mL/kg) medium (Sigma Aldrich, Germany).

### Experiment’s structure

**Fig. 1:**
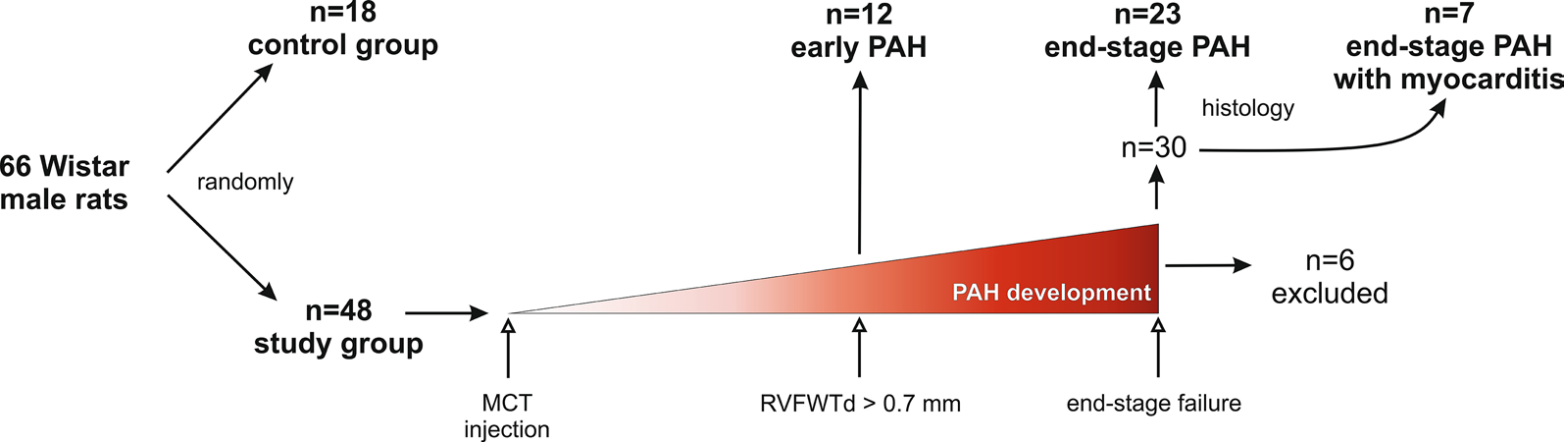
Flow chart demonstrating experiment’s structure. MCT: monocrotaline, PAH: pulmonary arterial hypertension, RVFWTd - end-diastolic right ventricular free wall thickness

Experiment’s structure is presented on Fig. 1. There were two endpoints in this study:

(1) early signs of PAH were defined as first morphological lesions of the right ventricle visible in in vivo echocardiography (RVFWTd > 0.7 mm) [12]. A total of 12 animals form the study group that first met this criterion were sacrificed.

(2) heart failure secondary to PAH (end-stage PAH) was defined as a clinical signs of right ventricular insufficiency up to end-stage circulatory and respiratory insufficiency, diagnosed when at least one of the following criteria occurred: [1] dyspnea, defined as increased respiratory effort and alternate respiratory motions of the rat’s thorax and the abdomen, [2] decreased temperature of the lower half of the body, the extremities and the tail, assessed subjectively during physical examination [3], cyanotic eyes [4], significantly decreased physical activity, lethargy. A total of 30 animals with heart failure were sacrificed.

The remaining rats from the study group that did not develop PAH (n = 4) or died in uncontrolled conditions (n = 2) were excluded from the study. As described in literature, the time of occurrence for hemodynamic (and next structural) signs of PAH after single monocrotaline administration in rats is diverse [13]. Due to this fact, rigid timeframe limiting maintenance of animals in the model was abandoned and only strong objective criteria (echocardiography and clinical signs) were used to allocate animals to specific study groups.

### Echocardiographic examination

Regularly performed transthoracic echocardiographic examinations were used to assess the dynamics of changes in pulmonary circulation and morphometric cardiac parameters. Examinations were performed using a transducer dedicated for echocardiographic examination of small animals (Mindray M7 with P12-4s, 4.2–11.0 MHz transducer, Mindray Bio-Medical Electronics Co., Shenzhen China) in all animals from both groups on the following days: +5, +10, +15, +18, +20, and then every 3 days and on the day the rats were euthanized. The echocardiography was performed on conscious animals (without any drug administration) who were immobilized manually in a supine position on the dorsum. To ensure the cooperation of animals, rats were subjected to extensive handling. The examination was performed with blinding—that is, the researcher was not aware of whether the animal belonged to the study group or the control group. In addition, digital recordings of the examinations were blindly assessed by two independent researchers to minimize human bias. End-diastolic left ventricular free wall thickness (LVFWTd) was assessed in the long-axis parasternal view, and end-diastolic right ventricular free wall thickness (RVFWTd) was assessed in the apical 4-chamber projection at a 10.0 MHz frequency and a rate of 114 frames/s. In addition, tricuspid annular plane systolic excursion (TAPSE) and pulmonary artery acceleration time normalized to cycle length (PAAT/CL) were measured according to standard guidelines [12, 14].

### Hemodynamic examination

All animals underwent invasive hemodynamic testing on the euthanasia day. The rats were placed in the dorsal position on the operating table and anesthetized with pentobarbital sodium (30 mg/kg body weight, Biowet, Poland), which was administered intraperitoneally. Animals were mechanically ventilated during the whole procedure using a pressure-controlled respirator and a mixture of air and oxygen. Anesthesia was maintained by additional bolus doses of pentobarbital sodium as needed. Lidocaine (20 mg/mL, B. Braun Melsungen AG, Germany) was used for local infiltration of the surgical sites. The chest cavity was opened by the left and right mini-thoracotomy in the 6th intercostal space. For the measurement of ventricular systolic and end-diastolic pressure, the heparinized 21G venous cannulas connected to a pressure recording system (Siemens SC 7000, Erlangen, Germany) were introduced simultaneously to the right and left ventricles via their apexes [13]. The pressure transducer was fixed to the operating table and set at the level of the animal’s heart. The values were registered from a stable signal with 300-s periods, and mean values were calculated as output values. After the procedure, animals were euthanized via an overdose of sodium pentobarbital (Biowet, Poland) which was administered intraperitoneally.

### Animal dissection

Directly after declaring termination of vital functions, the chest cavity was opened. The inferior vena cava and descending aorta were cannulated, all blood was removed, and infusion of the body using large volumes of Ringer’s solution (Fresenius Kabi, Germany) was conducted in order to clean the organs, including the myocardium, from protein material originating from the vascular bed. Next, the heart, along with proximal parts of main vessels, was dissected, blotted dry, and weighted using an electronic laboratory scale (Ohaus PA224C, Switzerland). Using a stereoscopic microscope, the pulmonary trunk and ascending aorta were dissected, and the diameters of their lumen were measured. Then, the atria and main vessels were separated from ventricles, the mid-diameters of the ventricles were measured, and then the ventricles were weighed en bloc. Next, muscle tissue of the left and right ventricle free wall and interventricular septum were completely separated from each other and the remaining heart structures and were then weighed. The whole wall thicknesses of the left and right ventricle free wall and interventricular septum were measured in their middle sectors. Linear measurements were performed using 0.03-mm precision electronic calipers (YT-7201 YATO, Poland). Small tissue samples from the left ventricle were fixed in 10% buffered paraformaldehyde solution.

### Histological analysis

**Fig. 2:**
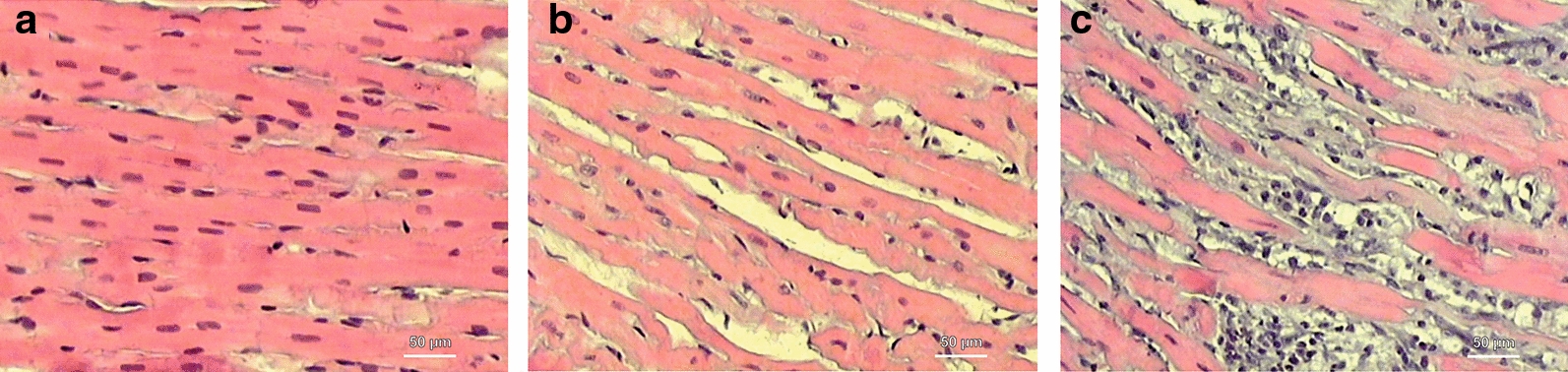
Histological cross-sections of left ventricle myocardium (hematoxylin and eosin) showing different stages of inflammatory cells infiltration to myocardium. **a** Lack of inflammation, **b** moderate inflammation, **c** severe inflammation

It has been proven that monocrotaline, apart from its pneumotoxicity responsible for PAH induction, also presents a direct cardiotoxic effect expressed by myocarditis [15]. Therefore, paraformaldehyde-fixed left ventricle samples were used to assess the microscopic structure of the myocardium and signs of possible inflammation. Samples were dehydrated in a series of alcohols. Then, they were cleared in xylene and embedded in paraffin blocks. Next, samples were cut into 5-µm thick sections and stained with hematoxylin and eosin (Sigma Aldrich, Germany) according to standard protocols [16]. The infiltration of inflammatory cells was assessed semi-quantitatively (0 = lack, 1 = low, 2 = moderate, 3 = high, 4 = severe) (Fig. 2), and samples with high and severe infiltration were defined as significant myocarditis samples.

### Statistical analysis

We performed statistical analyses using StatSoft STATISTICA 13.5 software for Windows (StatSoft Inc, Tulsa, OK). The data are presented as mean values with the corresponding standard deviations (SD) or percentages. All morphometric values were standardized to the rat’s body weight on the euthanasia day (morphometric value [in grams or mm]/body weight [in grams] * 0.01). The Shapiro–Wilk test was used to determine if the quantitative data were normally distributed. In order to compare values between groups, the analysis of variance (ANOVA) or non-parametric Kruskal–Wallis test was used. A detailed comparison was performed using Tukey’s post hoc analyses. An obtained p value of less than 0.05 was considered to be statistically significant.

## Results

### Basic groups characteristics

**Fig. 3:**
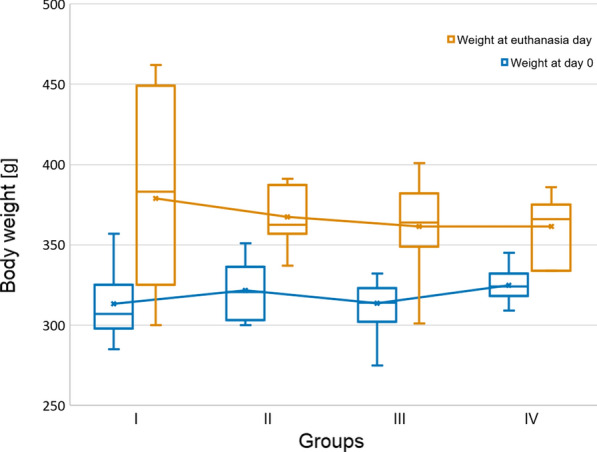
Body weight change over the time of the study (box whisker plots)

A histological analysis showed no significant myocarditis in non-PAH animals and in rats with early signs of PAH, but seven animals from the end stage-PAH subjects developed secondary to monocrotaline myocarditis (Fig. 2a–c). Therefore, four final groups were distinguished: I—non-PAH, control (n = 18); II—early PAH (n = 12); III—end-stage PAH (n = 23); and IV—end-stage PAH with myocarditis (n = 7) (Fig. 1), between which all measured parameters were compared. The mean experiment time (from day 0 to euthanasia) in the early PAH group was 20.0 ± 1.1 days, while it was 41.0 ± 8.4 days in the end stage-PAH group, 39.0 ± 5.8 days in the end-stage PAH with myocarditis group, and 27.2 ± 25.1 days in the control group. No statistically significant differences were observed between the starting body weight of the animals (at day 0), the body weight measured at euthanasia, or in body weight change over the time of study between all investigated groups (Table 1, Fig. 3).

**Table 1:** Changes of measured morphometric parameters over the course of pulmonary arterial hypertension (mean ± SD)

Parameter	I—non-PAH control	II—early PAH	III—end-stage PAH	IV—end-stage PAH with myocarditis	p-value ANOVA or Kruskal–Wallis test	Pairwise comparisons					
						p-value I vs. II	p-value I vs. III	p-value I vs. IV	p-value II vs. III	p-value II vs. IV	p-value III vs. IV
Body weight at 0 day (g)	313.2 ± 20.0	321.6 ± 18.2	313.7 ± 17.8	324.7 ± 11.3	0.368	ns	ns	ns	ns	ns	ns
Body weight at euthanasia day (g)	378.7 ± 58.0	367.4 ± 18.6	361.3 ± 28.3	361.4 ± 20.0	0.526	ns	ns	ns	ns	ns	ns
Body weight change (0 day—euthanasia day) (g)	65.6 ± 56.7	45.8 ± 6.9	47.5 ± 17.6	36.7 ± 15.7	0.209	ns	ns	ns	ns	ns	ns
Heart weight (g) [indexed]	1.04 ± 0.14 [0.28 ± 0.06]	1.01 ± 0.07 [0.28 ± 0.01]	1.21 ± 0.12 [0.34 ± 0.04]	1.36 ± 0.16 [0.38 ± 0.05]	*<0.001[< 0.001]*	0.962 [1.000]	*<0.001[0.004]*	*<0.001[< 0.001]*	*0.002[0.032]*	*<0.001[0.006]*	0.036 [1.000]
Ventricles weight (g) [indexed]	0.87 ± 0.10 [0.23 ± 0.04]	0.85 ± 0.05 [0.23 ± 0.01]	1.01 ± 0.08 [0.28 ± 0.03]	1.15 ± 0.11 [0.32 ± 0.04]	*<0.001[< 0.001]*	0.894 [1.000]	*<0.001[0.002]*	*<0.001[< 0.001]*	*<0.001[0.011]*	*<0.001[0.001]*	*0.005* [0.790]
RV free wall weight (g) [indexed]	0.16 ± 0.03 [0.04 ± 0.01]	0.19 ± 0.02 [0.05 ± 0.01]	0.35 ± 0.04 [0.10 ± 0.01]	0.39 ± 0.05 [0.11 ± 0.02]	*<0.001[< 0.001]*	0.425 *[< 0.001]*	*<0.001[< 0.001]*	*<0.001[< 0.001]*	*<0.001[< 0.001]*	*<0.001[< 0.001]*	0.140 [0.091]
LV free wall weight (g) [indexed]	0.37 ± 0.05 [0.10 ± 0.02]	0.35 ± 0.02 [0.09 ± 0.01]	0.28 ± 0.04 [0.08 ± 0.01]	0.32 ± 0.07 [0.09 ± 0.02]	*<0.001[< 0.001]*	0.736 [1.000]	*<0.001[< 0.001]*	0.701 [1.000]	0.053 [0.069]	1.000 [1.000]	0.103 [0.092]
LV free wall + IVS weight (g) [indexed]	0.68 ± 0.08 [0.18 ± 0.02]	0.64 ± 0.04 [0.17 ± 0.01]	0.65 ± 0.05 [0.18 ± 0.02]	0.70 ± 0.06 [0.19 ± 0.02]	0.071 [0.179]	ns	ns	ns	ns	ns	ns
IVS weight (g) [indexed]	0.29 ± 0.04 [0.08 ± 0.01]	0.31 ± 0.04 [0.08 ± 0.01]	0.35 ± 0.05 [0.10 ± 0.01]	0.41 ± 0.07 [0.11 ± 0.02]	*<0.001[< 0.001]*	0.751 [0.786]	*<0.001[< 0.001]*	*<0.001[< 0.001]*	*0.010[< 0.001]*	*<0.001[< 0.001]*	0.137 [0.049]
RV free wall thickness (mm) [indexed]	0.51 ± 0.14 [0.14 ± 0.04]	0.91 ± 0.14 [0.25 ± 0.04]	1.23 ± 0.25 [0.34 ± 0.09]	1.32 ± 0.20 [0.37 ± 0.07]	*<0.001[< 0.001]*	*0.002* [0.129]	*<0.001[< 0.001]*	*<0.001[< 0.001]*	*0.013[0.253]*	*0.002[0.188]*	0.820 [1.000]
LV free wall thickness (mm) [indexed]	2.37 ± 0.42 [0.63 ± 0.13]	2.54 ± 0.42 [0.67 ± 0.10]	1.98 ± 0.41 [0.55 ± 0.13]	2.25 ± 0.50 [0.62 ± 0.13]	*0.005* [0.068]	0.280 [ns]	*0.004* [ns]	0.543 [ns]	*0.023* [ns]	0.193 [ns]	0.380 [ns]
IVS thickness (mm) [indexed]	1.43 ± 0.33 [0.39 ± 0.14]	1.98 ± 0.36 [0.54 ± 0.09]	1.97 ± 0.37 [0.55 ± 0.13]	2.09 ± 0.49 [0.59 ± 0.17]	*<0.001[0.003]*	*0.021[0.014]*	*0.020[0.010]*	*0.008[0.019]*	1.000 [1.000]	0.950 [1.000]	0.243 [0.893]
RV diameter (mm) [indexed]	3.43 ± 0.78 [0.89 ± 0.23]	3.76 ± 0.92 [1.02 ± 0.25]	4.39 ± 0.89 [1.22 ± 0.25]	5.04 ± 0.71 [1.39 ± 0.15]	*<0.001 [< 0.001]*	0.865 [0.547]	*0.013[< 0.001]*	*0.005[< 0.001]*	0.456 [0.190]	*0.032[0.018]*	0.475 [0.320]
LV diameter (mm) [indexed]	4.77 ± 0.70 [1.27 ± 0.21]	4.81 ± 0.65 [1.31 ± 0.18]	3.03 ± 0.69 [0.83 ± 0.18]	2.96 ± 0.47 [0.82 ± 0.09]	*<0.001 [< 0.001]*	0.876 [0.897]	*<0.001[< 0.001]*	*<0.001[< 0.001]*	*<0.001[< 0.001]*	*<0.001[< 0.001]*	0.998 [0.993]
Pulmonary trunk diameter (mm) [indexed]	2.35 ± 0.36 [0.63 ± 0.12]	2.34 ± 0.29 [0.64 ± 0.08]	3.11 ± 0.42 [0.86 ± 0.12]	3.08 ± 0.27 [0.85 ± 0.04]	*<0.001[< 0.001]*	1.000 [0.992]	*<0.001[< 0.001]*	*<0.001[< 0.001]*	*<0.001[< 0.001]*	*0.002[0.002]*	0.999 [0.994]
Aorta diameter (mm) [indexed]	2.29 ± 0.21 [0.62 ± 0.10]	2.50 ± 0.33 [0.68 ± 0.09]	2.51 ± 0.25 [0.70 ± 0.10]	2.48 ± 0.40 [0.69 ± 0.12]	0.069 [0.077]	ns	ns	ns	ns	ns	ns

IVS: interventricular septum; LV: left ventricle; PAH: pulmonary arterial hypertension; RV: right ventricle

Statistically significant p-values are given in italics

### Echocardiographic and hemodynamic parameters

**Fig. 4:**
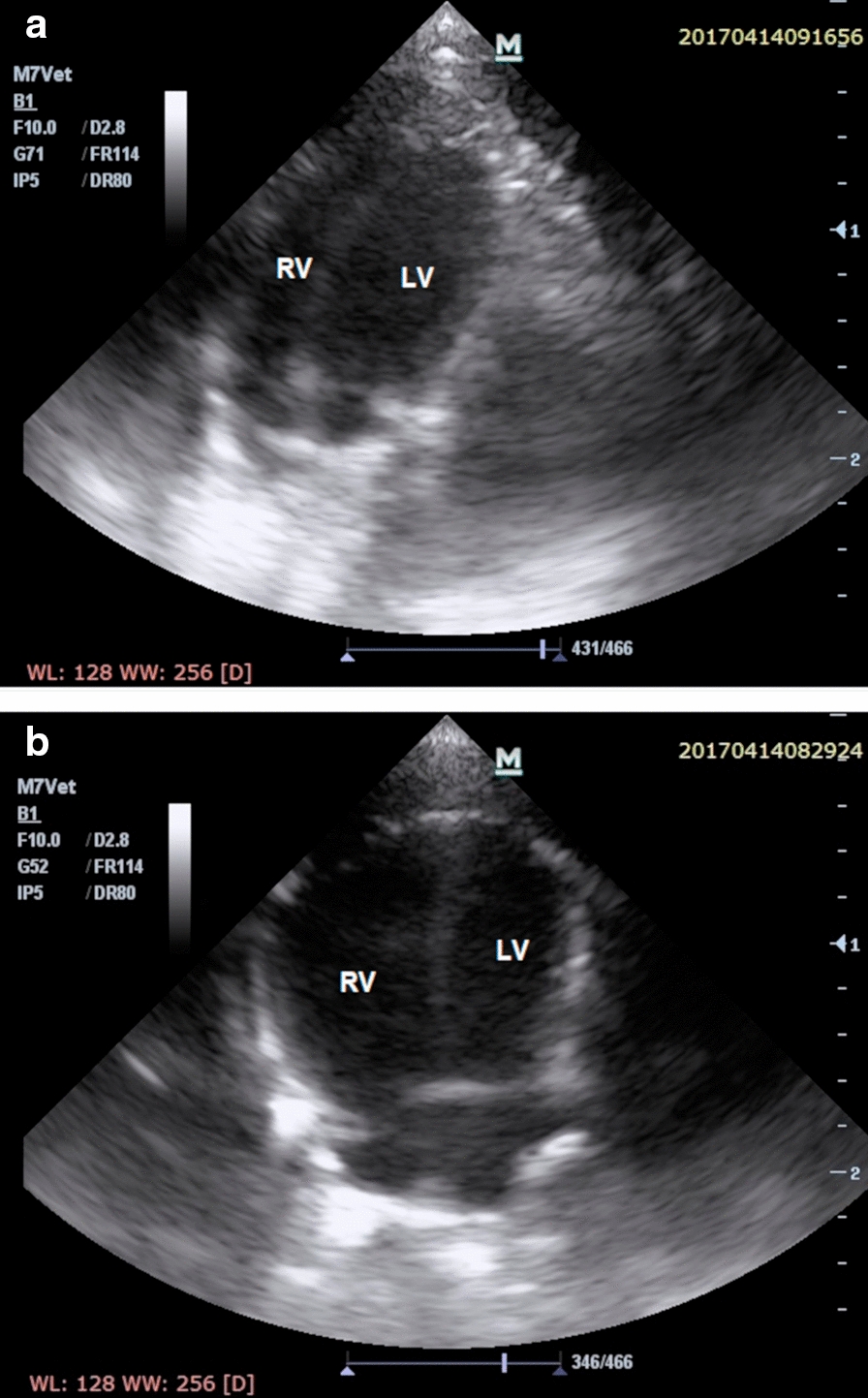
Transthoracic echocardiogram images in the apical 4-chamber end-diastolic projection showing **a** normal heart of the rat from the control group and **b** failing heart with end-stage monocrotaline-induced pulmonary arterial hypertension and right ventricular hypertrophy in rat. LV: left ventricle, RV: right ventricle

Table 2 presents between-groups differences in echocardiographic and hemodynamic parameters measured at on the euthanasia day. Significant thickening of the right ventricle free wall, TAPSE reduction, as well as a decrease in PAAT/CL were observed over the course of PAH (Fig. 4). No differences in left ventricle free wall thickness (LVFWTd) measured in echocardiography was observed between groups.

**Table 2:** Echocardiographic and hemodynamic parameters measured at euthanasia day (mean ± SD)

Parameter	I—non-PAH control	II—early PAH	III—end-stage PAH	IV—end-stage PAH with myocarditis	p-value ANOVA or Kruskal–Wallis test	Pairwise comparisons					
						p-value I vs. II	p-value I vs. III	p-value I vs. IV	p-value II vs. III	p-value II vs. IV	p-value III vs. IV
LVFWTd (mm)	0.20 ± 0.04	0.21 ± 0.03	0.19 ± 0.05	0.21 ± 0.03	0.569	ns	ns	ns	ns	ns	ns
RVFWTd (mm)	0.61 ± 0.05	0.78 ± 0.04	0.95 ± 0.20	0.88 ± 0.33	*<0.001*	*<0.001*	*<0.001*	*0.002*	*0.007*	0.305	1.000
TAPSE (mm)	1.11 ± 0.64	1.02 ± 0.13	0.72 ± 0.16	0.71 ± 0.14	*<0.001*	1.000	*0.010*	0.129	*<0.001*	*<0.001*	1.000
PAAT/CL (mm/s)	0.23 ± 0.15	0.21 ± 0.05	0.14 ± 0.06	0.12 ± 0.04	*<0.001*	1.000	*0.007*	*0.017*	0.127	0.086	1.000
LV systolic pressure (mmHg)	90.09 ± 15.90	80.50 ± 3.00	46.94 ± 14.65	43.00 ± 9.56	*<0.001*	1.000	*<0.001*	0.004	*0.012*	*0.040*	1.000
LV diastolic pressure (mmHg)	9.00 ± 4.86	7.50 ± 2.08	6.94 ± 4.02	3.50 ± 1.91	0.073	ns	ns	ns	ns	ns	ns
RV systolic pressure (mmHg)	20.20 ± 6.60	28.50 ± 5.20	45.10 ± 12.83	49.67 ± 19.91	*<0.001*	*<0.001*	*<0.001*	*0.006*	*0.001*	*0.011*	1.000
RV diastolic pressure (mmHg)	4.40 ± 2.55	8.00 ± 2.00	6.00 ± 3.89	6.67 ± 4.68	*0.012*	*0.008*	0.748	1.000	0.161	0.508	1.000

LV: left ventricle; LVFWTd: end-diastolic left ventricular free wall thickness; PAAT/CL: pulmonary artery acceleration time normalized to cycle length; PAH: pulmonary arterial hypertension; RV: right ventricle; RVFWTd: end-diastolic right ventricular free wall thickness; TAPSE: tricuspid annular plane systolic excursion

Statistically significant p-values are given in italics

### Hemodynamic parameters

**Fig. 5:**
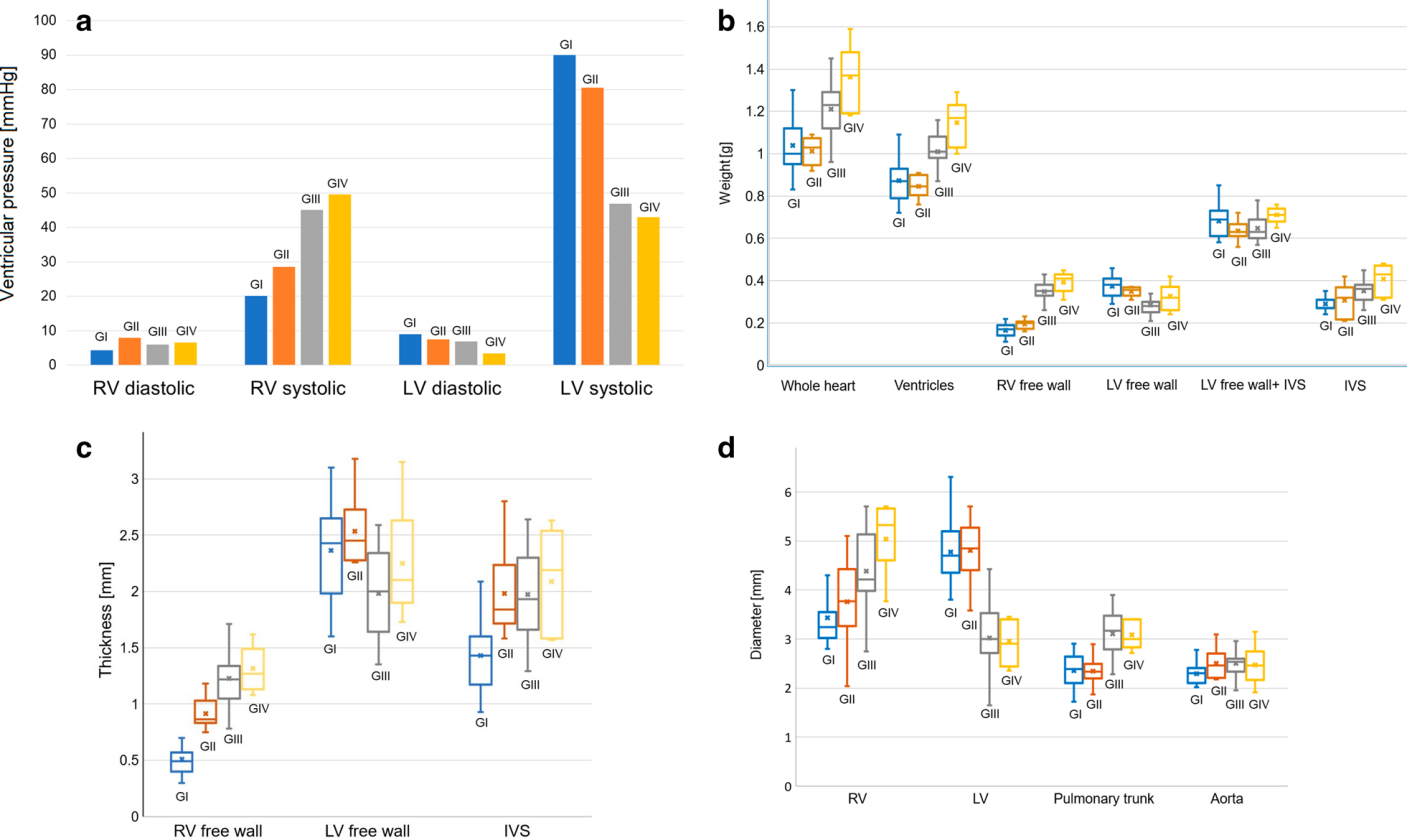
**a** Between groups hemodynamic parameters differences. **b** Measured weights of the heart structures with between-group comparisons (box whisker plots). **c** Measured thicknesses of the heart structures with between-group comparisons (box whisker plots). **d** Measured diameters of the heart structures with between-group comparisons (box whisker plots). IVS: interventricular septum, LV: left ventricle, RV: right ventricle

Obtained hemodynamic parameters indicated development of the pulmonary hypertension in the II, III, and IV groups (significant increase of right ventricle systolic pressure compered to controls). Also, a significant change was observed in the left ventricle systolic pressure, which gradually decreased with the development of PAH (Fig. 5a).

### Morphometric heart parameters at sacrifice

Significant differences were observed in total heart weight as well as in the weight of the ventricles, which was greater in groups with end-stage PAH (III and IV group) than in controls and the early PAH group (p < 0.001, Table 1, Fig. 5b).

Multidimensional changes in the structure of the right ventricle were observed (Table 1). In particular, a gradual thickening of the right ventricle free wall was observed over the course of PAH (p < 0.001), with an estimated percentage increase of 241.2% (I group vs. III group) (Fig. 5c). The same trend was observed for the right ventricle free wall weight, which was significantly heavier in the end-stage PAH (III and IV) groups than in controls (p < 0.001), but no statistically significant difference was observed when comparing controls and early PAH animals (p = 0.425), though significance was achieved after indexation to whole body weight (p < 0.001) (Fig. 5b). Moreover, significant dilatation of the right ventricle was observed in the end-stage PAH groups (III and IV group) (Table 1, Fig. 5d).

A significant reduction in the left ventricle free wall thickness was observed when comparing non-PAH or early PAH with end-stage PAH without myocarditis (Table 1, Fig. 5c). The interventricular septum thickness was significantly larger in monocrotaline-induced PAH animals than in the control group (p < 0.001, Table 1, Fig. 5c). Measurements of the left ventricle with interventricular septum weight revealed no significant differences between the studied groups in this parameter (p = 0.071, Fig. 5b). The interventricular septum weight significantly increased over the PAH development (p < 0.001, Table 1, Fig. 5b).

A significant trend in the left ventricle free wall weight reduction was observed over the course of PAH, which was the greatest when comparing non-PAH controls with end-stage PAH animals (I group vs. III group, p < 0.001, Fig. 5b), with an estimated percentage reduction of 24.3%. The calculated right/left ventricle free wall weight ratio gradually increased over PAH stages (I group: 0.44 ± 0.06, II group: 0.56 ± 0.09, III group: 1.25 ± 0.25, IV group: 1.28 ± 0.39; p < 0.001). The reduction of the left ventricle diameter was observed in rats with end-stage PAH both with and without myocarditis (p < 0.001, Fig. 5d).

The pulmonary trunk diameter was significantly larger in the end-stage PAH and end-stage PAH groups with inflammation than in controls or in early PAH groups (Table 1, Fig. 5d). In addition, the aorta diameter (both native value and indexed to body weight) did not differ significantly between the study groups (Table 1, Fig. 5d).

## Discussion

PAH is a group of heterogeneous rare diseases (prevalence estimated around 30-50/1000,000) that is characterized by elevated pulmonary arterial resistance, leading to end-stage heart failure, and has a significant impact on survival [17–19]. PAH is mainly diagnosed at its advanced stage and is ultimately a fatal disorder. In the natural history of idiopathic PAH the estimated median survival time of patients is 2.8 years [20]. Although general knowledge about the natural course of the PAH has increased importantly in the past few decades, PAH continues to be a devastating disease with poor survival, decreased patient quality of life, and high economic burden [21]. Numerous efforts are being made to deeply explore the pathophysiology of PAH and to discover factors influencing the prognosis or modulating the course of the disease, thus promoting patient survival in the future. Nevertheless, there are still many unanswered questions, especially those regarding altered morphology of the heart in PAH. Only a few studies have investigated specific cardiac morphometric deviations during PAH, mainly focusing on patients with end-stage right ventricular failure [22]. Meanwhile, knowledge of early organ changes may facilitate prompt detection of PAH and effective monitoring of disease progress from its very beginning, which may delay harmful progress of heart remodeling. Although the function of the heart and its basic dimensions (e.g., wall thickness, chamber dimensions) are easily achievable in vivo by available imaging techniques (e.g., echocardiography, computed tomography, magnetic resonance imaging), the assessment of the weight of individual heart elements is still affected by significant measurement bias [23]. For this reason, animal models that allow for direct measurements of the heart during autopsy at subsequent stages of the disease should be used [24].

Our study provides cardiac morphometric characteristics at different stages of monocrotaline-induced PAH and confirms previously reported right-ventricle-related changes, which include right ventricle myocardium hypertrophy and chamber dilatation that gradually progress from the onset of elevated pulmonary arterial pressure. In early PAH, the right ventricle retains abilities to adapt to increased afterload (pressure overload) with increased contractility and slight increases in right ventricle wall thickness and chamber dimensions, allowing for a stable stroke volume [25]. With exacerbation of the disease, the compensatory right ventricular remodeling cannot remain matched to the highly increased afterload, and its serious dysfunction leads to end-stage heart failure. The mechanisms responsible for right heart remodeling and insufficiency are fairly well understood and include neurohumoral activation, apoptosis, capillary loss, expression of inflammatory mediators, oxidative stress, ischaemia, and metabolic shifts, with variable fibrosis and hypertrophy [26, 27].

The left ventricle has thus far played a secondary role in PAH studies and clinical practice. To illustrate this neglected phenomenon, the phrase “the forgotten left ventricle” was created [7]. As shown in the current study, the function of the left ventricle was observed to be impaired, which was expressed by significant left ventricle systolic pressure reduction (non-PAH: 90.09 ± 15.90 mmHg vs. end-stage PAH: 46.94 ± 14.65 mmHg; p < 0.001, Table 2). Apart from the functional impairment, there is a significant 25% reduction of the left ventricle free wall mass in end-stage PAH compared to healthy subjects. Moreover, both the left ventricle free wall thickness and the diameter of the left ventricle were significantly reduced in the end-stage PAH group (Table 1).

Although left ventricle atrophy was previously described in a rat model of monocrotaline induced PAH by Hardziyenka et al. our study adds to the current understanding of PAH induced changes in heart morphology [6]. First the left ventricular myocardium deterioration and failure were observed only at later stages of the PAH and were preceded by a significant remodeling of the right ventricle. Second, we have found that monocrotaline induced myocarditis may significantly change our understanding of its effects on cardiac morphometry. The molecular processes (genomic and proteomic) underlying left heart ventricle remodeling over the course of PAH remain unknown. In particular, there is no knowledge regarding the mechanisms of left heart ventricle myocardium mass loss, and this topic has been completely avoided by researchers until recently [6, 28]. One of the proposed mechanisms of left ventricle atrophy includes a decrease in the initial load of the left heart part (under-filling of the left ventricle, hemodynamic stress). Another possible mechanism of left ventricle mass loss over the course of PAH includes hypoxia and ischaemia of the myocardium, resulting from right ventricle heart failure (metabolic stress) [29]. It is assumed that the left ventricle mass loss over the course of pulmonary hypertension results more from a mass decrease and reduction in size of the cardiomyocytes themselves (atrophy) than the decrease in their number (apoptosis) [28].

The monocrotaline is an 11-membered macrocyclic pyrrolizidine alkaloid derived from the seeds of the *Crotalaria spectabilis* plant [30]. A monocrotaline model of PAH in rats is a well-researched and widely used experimental model. After intraperitoneal administration of a single dose, monocrotaline is activated to its toxic form (dehydromonocrotalin) in hepatic P450 3A cytochrome. Pneumotoxic alkaloid damages the endothelium of arterial pulmonary vessels and causes its pathological remodeling, which results in increased resistance of pulmonary arteries, development of pulmonary hypertension, right ventricular overload, hypertrophy of the right ventricle myocardium with its subsequent insufficiency, and heart failure. Despite its numerous disadvantages, this model remains referential in terms of other experimental models [31]. Among other preclinical models of PAH, the monocrotaline animal model offers the advantage of mimicking several key aspects of human PAH, including vascular remodeling, proliferation of smooth muscle cells, endothelial dysfunction, upregulation of inflammatory cytokines, and right ventricle failure, which is the cause of subsequent changes in the whole circulatory system [32]. Nevertheless, as shown in this study, 14.6% of all monocrotaline injected rats have developed considerable left ventricle myocarditis, which was observed in animals with end-stage PAH but not in early PAH. Our morphometric analyses showed that the group with myocarditis differed from the non-myocarditis end-stage PAH group in several aspects, including increased weight and thickness of all measured structures. These changes may be explained by significant inflammatory infiltration, which causes an increase of the size and weight of the structures. Though these findings were mainly statistically insignificant, the described phenomenon should not be overlooked in future studies using monocrotaline-induced PAH models, because myocarditis may considerably affect the results of observations [33].

Our purely morphometric study is not without limitations. First, results of hemodynamic measurements may be affected by mechanical ventilation and pneumothorax developed due to micro-thoracotomy, nevertheless the conditions of study were the same for all animals and general trends in the ventricular pressure should not be affected. Furthermore, no tissue or molecular mechanisms of observed changes were studied; therefore, these should be the subject of further research. Although data presented in this study should be cautiously extrapolated to human subjects, the monocrotaline-induced PAH experimental model is a generally accepted model to study PAH and heart morphometric changes observed in animals and should be similar to those in humans [31].

## Conclusions

Multidimensional morphometric changes of heart structures occur over the course of PAH, and macroscopic changes progress gradually. Early PAH changes include only right ventricle free wall and interventricular septum thickening, while end-stage PAH changes include right ventricle free wall thickening and weight increase, right ventricle dilatation, left ventricle free wall thinning and weight reduction, left ventricle stricture, interventricular septum thickening and weight increase, and pulmonary trunk dilatation. In summary, right ventricle functional failure and morphometric changes develop gradually from the early stage of the PAH, while left ventricle changes develop during the end stages of the disease. Knowledge on the sequence of changes over the PAH stages will allow to determine high-risk PAH patients through a screening process and may facilitate the decision to escalate PAH targeted therapies.

## Data Availability

The datasets used and/or analysed during the current study are available from the corresponding author on reasonable request.

## Abbreviations

PAH: Pulmonary arterial hypertension
LVFWTd: End-diastolic left ventricular free wall thickness
RVFWTd: End-diastolic right ventricular free wall thickness
TAPSE: Tricuspid annular plane systolic excursion
PAAT/CL: Pulmonary artery acceleration time normalized to cycle length
